# Calculating mutual information for spike trains and other data with distances but no coordinates

**DOI:** 10.1098/rsos.140391

**Published:** 2015-05-13

**Authors:** Conor Houghton

**Affiliations:** Department of Computer Science, University of Bristol, Merchant Venturers Building, Woodland Road, Bristol BS8 1UB, UK

**Keywords:** spike trains, information theory, mutual information

## Abstract

Many important data types, such as the spike trains recorded from neurons in typical electrophysiological experiments, have a natural notion of distance or similarity between data points, even though there is no obvious coordinate system. Here, a simple Kozachenko–Leonenko estimator is derived for calculating the mutual information between datasets of this type.

## 2. Introduction

This article describes a simple formula for calculating the mutual information between random variables where one or both of the variables take values in a metric space. This is relevant to neuroscience because electrophysiological data, whether spike trains from single neurons or collections of spike trains from a population of neurons, can be naturally considered to take values in a metric space [1,2]. It is, in turn, useful to be able to calculate information theory quantities for these data as part of an investigation into effective coding theories of neurodynamics or as a tool for quantifying the relationship between the activity of different neurons or different neuronal populations. However, the relevance is not limited to spike trains, it extends to other electrophysiological data types, such as calcium or electroencephalogram traces. Indeed, non-coordinate metrics or similarity measures are also used, for example, in genetics and biochemistry [3,4], in image analysis [5] and in information retrieval [6].

The aim here is to address two difficulties associated with estimating mutual information on metric spaces. The first difficulty is that information theory is most typically applied to problems where the data are either discrete or take values in an integrable manifold. The second difficulty is that many approaches to estimating information theory quantities can demand an unrealistically large amount of data. The second of these difficulties has been addressed in the past by the Kozachenko–Leonenko estimator [7–10], but, in line with the first difficulty, this estimator is derived specifically for an integrable manifold or using a local effective dimension. The aim here is to provide a simple approach to a Kozachenko–Leonenko estimator which applies to metric spaces. With this extension, mutual information can be calculated for the broad class of important data where there is a similarity measure, but no coordinates.

The particular application which motivates this article is the problem of calculating information theory quantities for spike trains. Three different types of neuroscience experiment could be considered typical. In the first, the activity of a neuron or group of neurons is recorded from the brain of an anesthetized or restrained animal while that animal is being presented with a series of stimuli. The challenge is to estimate the mutual information between the stimulus and the neuronal activity during the presentation. In the second, neuronal activity is recorded while an animal is moving freely in an arena and the mutual information is to be estimated between the position of the animal at a given time and the neuronal activity in a temporal window centred on that time. In the third example, the mutual information is to be estimated between temporal slices of the spike trains produced by different neurons so that this can be used to measure the relationship between those neurons, for example, at different times during development.

Estimating mutual information is not straightforward in any of these examples because there is no obvious coordinate system for describing neuronal activity. One approach to solving this difficulty is to discretize the spike trains, turning individual fragments of spike train into sequences of ones and zeros with each bit accounting for the presence or absence of a spike in a corresponding time slot [11]. However, the amount of possible words is huge and so this approach is bedevilled by the large amount of data it requires. One response to this problem is to exploit what is sometimes called the birthday problem and to look at coincidences [12,13]. However, the proximity structure of the space of spike trains is poorly approximated by examining the coincidence whereby two spike trains are discretized to the same sequence of ones and zeros. Here, it is proposed that, instead, one of the many metrics or similarity measures on the space of spike trains be used to define proximity [14–20].

## 3. Method

Two simple formulae for mutual information are presented in this article, one for the mutual information between a discrete space and a metric space and one for the mutual information between two metric spaces. These two formulae are intended to cover, respectively, the first and the second and third neuroscientific examples described above. There are two main steps to deriving these formulae. Firstly, probabilities are estimated using a simplified version of the Kozachenko–Leonenko approach [7–10]; this estimate of probability involves terms that depend on the volumes. In the second step, these volumes are estimated using the probability distribution as a measure.

### 3.1 A formula for the entropy

Consider estimating entropy for a random variable *X* which takes values in a space X with probability mass density *p*_*X*_(*x*). Given a set of *N* outcomes, {*x*_1_,*x*_2_,…,*x*_*N*_}, the entropy is estimated by

3.1 $$H(X)\approx -\frac{1}{N}\sum_{i=1}^{N}\log_{2}p_{X}(x_{i}).$$

The problem here is how to calculate this quantity when *p*_*X*_(*x*) is not known. To do this, the approach given in [7,9] is followed in spirit but modified to avoid any quantities that rely on coordinates; the aim is to derive a formula for metric spaces. This will require that a region *B*(*x*_*i*_,*V*) with volume *V* is chosen around each data point; this region will ultimately be specified using the metric, but for now it is supposed only that there is a such a region for each data point.

Now, consider the probability *P*_*k*_(*x*_*i*_) that the region *B*(*x*_*i*_,*V*) contains precisely *k* points; it is

3.2 $$P_{k}(r_{i})=\left(\begin{array}{c} N\\ k \end{array}\right)F_{i}^{k}{\left(1-F_{i}\right)}^{N-k},$$

where *F*_*i*_ is the probability mass contained in *B*(*x*_*i*_,*V*). This means that

3.3 $$\langle k\rangle =NF_{i}.$$

This quantity can be estimated from the data

3.4 $$\langle k\rangle \approx \#[B(x_{i},V)],$$

where #[*B*(*x*_*i*_,*V*)] denotes the number of data points in *B*(*x*_*i*_,*V*); that is for any B⊆X

3.5 $$\#[B]=|B\cap \{x_{1},x_{2},\ldots ,x_{N}\}|.$$

This means that

3.6 $$NF_{i}\approx \#[B(x_{i},V)].$$

Now, the probability mass function is approximated by assuming that it is constant in the ball

3.7 $$F_{i}\approx Vp_{X}(x_{i}).$$

This means

3.8 $$NVp_{X}(x_{i})\approx \#[B(x_{i},V)]$$

so

3.9 $$\log_{2}p_{X}(x_{i})\approx \log_{2}\#[B(x_{i},V)]-\log_{2}N-\log_{2}V$$

or

3.10 $$H(X)\approx \log_{2}N+\log_{2}V-\frac{1}{N}\sum_{i=1}^{N}\log_{2}\#[B(x_{i},V)].$$

This assumption is the same as the one used in [7,9]; in [9], some care is given to justifying this as an approximation; here, though, it is introduced only with the general justification that the variation in *p*_*X*_(*x*) should be modest if the region spanned by *B*(*x*_*i*_,*V*) is small.

The formula for the entropy, equation (3.10), is similar in spirit to the one given in [7,9]. However, it is not identical, and is, in fact, simpler, because here the probability is estimated using the expected number of points in a ball rather than the size of the ball that contains a given number of points; the latter requires the trinomial, as opposed to the binomial, expansion. Also, of course, the approach here is chosen because it makes it possible to avoid quantities that are only defined on integrable manifolds.

### 3.2 Estimating the volume

The problem with this is that there may not be an obvious measure. Certainly in the case of spike trains there are no good coordinates and so there is no way to calculate the volume of a region based on the usual sort of coordinate-based measure. However, a probability distribution always defines a measure: the volume of a region can be defined as being equal to the probability mass it contains,

3.11 vol B=P(x∈B).

This volume can be estimated from the data

3.12 $$\text{vol}\;B\approx \frac{\#[B]}{N}.$$

This gives a trivial estimate of the entropy: differential entropy is zero if the probability distribution is used as the measure and the approximation used here is exact in this case: if *V* =*h*/*N* for some integer *h*≤*N*

3.13 $$H(X)\approx \log_{2}N+\log_{2}\frac{h}{N}-\frac{1}{N}\sum_{i=1}^{N}\log_{2}h=0$$

as #[*B*(*x*_*i*_,*h*/*N*)]=*h* by definition. Thus, using the probability as a measure for estimating information theory quantities would be useless if the aim was to estimate entropy. In fact, the entropy is a measure-dependent quantity whose value changes if the measure is changed. This is perhaps less important when a particular relevant coordinate system distinguishes a measure, but generally the significance of the entropy is not clear. The mutual information, however, does not suffer from this problem, its value is independent of the measure used. Furthermore, from the perspective of the approach taken in this article, it involves more than one distribution which means that one distribution can be used to give a measure while calculating estimates for the other distributions.

Here, two cases will be considered: in the first case one random variable is discrete and the other takes its values in a metric space; in the second case both random variables take values in metric spaces. In addition, an estimate is derived for the Kullback–Leibler (KL) divergence between two distributions over the same metric space.

### 3.3 The mutual information where one random variable is discrete

In many electrophysiological experiments, stimuli from a discrete corpus are presented repeatedly while spike trains are recorded. In this situation, the stimuli are represented by a discrete random variable and the response by a random variable taking values in a metric space. This situation is considered here; the situation is more general than the neuroscientific application, but, for convenience, the terminology is based on that application.

Let S be a discrete set representing the stimuli and let R be a set of responses, which may be spike trains or sets of spike trains recorded from multiple neurons. For simplicity, consider the situation where each element of S is presented an equal number of times, *n*_t_. Let $n_{s}=|S|$ be the number of stimuli, so the total number of data points is *N*=*n*_t_*n*_S_.

The metric on R is used to define the regions that are required around the data points. For a point *r* in R define an open ball

3.14 $$B_{\epsilon }(r)=\{t\in R:d(r,t)<\epsilon \}.$$

Next, let *B*(*r*,*V*) be the open ball *B*_*ϵ*_(*r*) with *ϵ* chosen so that *B*_*ϵ*_(*r*) has volume *V* . The total probability *p*_*R*_(*r*) will be used as a measure and the volume *V* fixed at *V* =*h*/*N* for some *h*≤*N*. This means that *B*(*r*,*h*/*N*) is the open ball around *r* which contains *h* points.

With this measure *H*(*R*)=0. The same measure can be used for the conditioned probabilities; that is, for calculating *H*(*R*|*S*=*s*) using the conditioned probability *p*_*R*|*S*=*s*_(*r*). Hence,

3.15 $$\begin{array}{rl} H(R|S=s) & \approx \log_{2}n_{t}+\log_{2}\frac{h}{N}-\frac{1}{n_{t}}\sum_{i=1}^{N}\log_{2}\#\left[B\left(r_{i},\frac{h}{N}\right)\right]\\ & \approx -\log_{2}n_{s}+\log_{2}h-\frac{1}{n_{t}}\sum_{i=1}^{N}\log_{2}\#\left[B\left(r_{i},\frac{h}{N}\right)\right]. \end{array}$$

Thus, averaging over s∈S

3.16 $$\begin{array}{rl} I(R;S) & \approx \log_{2}n_{s}-\log_{2}h+\frac{1}{N}\sum_{i=1}^{N}\log_{2}\#\left[B\left(r_{i},\frac{h}{N}\right)\right]\\ & \approx \frac{1}{N}\sum_{i=1}^{N}\log_{2}\frac{n_{s}\#[B(r_{i},h/N)]}{h}. \end{array}$$

This formula is the same as the one proposed in [21]. However, it is derived there in a convoluted way which leaned heavily on intuition, whereas here the derivation is straightforward and can be easily extended to the case where both S and R are metric spaces. It is pointed out there that although the estimate given in [9] is derived using coordinate-based quantities, it can be used to give a formula that applies in this case. Numerical experiments in [21] comparing the two formulae gave very similar results.

### 3.4 The mutual information where both random variables take values in metric spaces

If S and R are both metric spaces, the marginal probability mass functions *p*_*R*_(*r*) and *p*_*S*_(*s*) give volume measures and with these measures *H*(*R*)=*H*(*S*)=0. These same measures also induce a measure on R×S, the space where (*R*,*S*) takes its values. In other words, *p*_*R*,*S*_(*r*_*i*_,*s*_*i*_) is estimated by considering regions around (*r*_*i*_,*s*_*i*_) whose volumes are calculated using the measure *p*_*R*_(*r*)*p*_*S*_(*s*) induced from the marginal spaces R and S. Thus, a square is used to define the regions:

3.17 $$S\left(r_{i},s_{i},\frac{h_{1}}{N},\frac{h_{2}}{N}\right)=\left\{(r,s)\in R\times S:r\in B_{R}\left(r_{i},\frac{h_{1}}{N}\right),\;s\in B_{S}\left(s_{i},\frac{h_{2}}{N}\right)\right\},$$

where *h*_1_/*N* and *h*_2_/*N* are the volumes chosen for R and S. Now, under the induced measure

3.18 $$\text{vol}\;S\left(r_{i},s_{i},\frac{h_{1}}{N},\frac{h_{2}}{N}\right)=\text{vol}\;B_{R}\left(r_{i},\frac{h_{1}}{N}\right)\text{vol}\;B_{S}\left(s_{i},\frac{h_{2}}{N}\right)\approx \frac{h_{1}h_{2}}{N^{2}}$$

so

3.19 $$I(R;S)\approx \frac{1}{N}\sum_{i=1}^{N}\log_{2}\frac{N\#[S(s_{i},r_{i},h_{1}/N,h_{2}/N)]}{h_{1}h_{2}}.$$

Thus the mutual entropy depends on #[*S*(*s*_*i*_,*r*_*i*_,*h*_1_/*N*,*h*_2_/*N*)] which counts the number of stimulus–response pairs (*s*,*r*), where *s* is one of the *h*_1_ points closest to *s*_*i*_ and *r* is one of the *h*_2_ points closest to *r*_*i*_.

It is instructive to consider what happens when the two variables are independent. By definition, *B*_*R*_(*r*_*i*_,*h*_1_/*N*) contains *h*_1_ points out of *N*; as *R* and *S* are independent this means the average number of points in *B*_*S*_(*s*_*i*_,*h*_2_/*N*) which are also in *B*_*R*_(*r*_*i*_,*h*_1_/*N*) is *h*_1_*h*_2_/*N* so

3.20 $$\frac{N\#[S(s_{i},r_{i},h_{1}/N,h_{2}/N)]}{h_{1}h_{2}}\approx 1.$$

The formula includes two integer parameters, *h*_1_ and *h*_2_, which need to be chosen; large values for these parameters reduce the accuracy of the approximation in equation (3.7) where the probability is taken as constant throughout the region, whereas taking a smaller value reduces the accuracy of the approximation in equations (3.6) and (3.12) where the mean or volume is estimated by counting.

### 3.5 The Kullback–Leibler divergence on a metric space

The approach described in this article also gives an estimate for the KL divergence. Consider two random variables *R* and *S* on a metric space X with probability mass functions *p*_*R*_(*x*) and *p*_*S*_(*x*). If {*r*_1_,*r*_2_,…,*r*_*M*_} and {*s*_1_,*s*_2_,…,*s*_*N*_} are sampled from *R* and *S* then the KL divergence is estimated by

3.21 $$d(R|S)\approx \frac{1}{M}\sum_{i=1}^{M}\log_{2}\frac{p_{R}(r_{i})}{p_{S}(r_{i})}.$$

Now, as before

3.22 $$MVp_{R}(r_{i})\approx \#[B(r_{i},V)].$$

However, in this case, the other distribution on the same space is used to measure the volume. If the volume is chosen as *h*/*N*, then *B*(*r*_*i*_,*h*/*N*) is the ball around *r*_*i*_ chosen to be large enough to include *h* points from {*s*_1_,*s*_2_,…,*s*_*N*_} and #[*B*(*r*_*i*_,*h*/*N*)] is the number of points from {*r*_1_,*r*_2_,…,*r*_*M*_} in the ball. The usual formula then gives

3.23 $$d(R|S)\approx \frac{1}{M}\sum_{i=1}^{M}\log_{2}\frac{N\#[B(r_{i},h/N)]}{Mh}.$$

It is easy to check that this formula gives an alternative derivation of the formula above for the mutual information between two random variables on metric spaces.

## 4. Conclusion

The formula derived here is very simple and is derived without any reference to coordinates on the sample space. It is intended that this demonstrates that the Kozachenko–Leonenko approach applies to metric spaces. The Kozachenko–Leonenko formula presented in [9] relies on a manifold structure in its derivation, but in its final form it is also applicable to metric spaces. It seems unlikely that the performance of the estimates here will be different from the performance of that formula.

In the estimates here, the distance or similarity measure is only required to order the points. Although electrophysiological data are used as a paradigmatic example, the formula can be applied to any pair of random variables taking values in metric spaces or indeed any space with a similarity or distance function suitable for defining regions surrounding each data point. As such it could be used in a straightforward way to calculate, for example, the mutual information between local field potentials and spike trains, or between the position of an animal in a maze and a neuronal population response, or, indeed, between two collections of images, or between images and text.

The estimate provided here relies on an approximation in which probabilities are replaced by counting how many data points fall within a region in space. This makes sound intuitive sense, but it has not been proved here that the estimate is a good one. Indeed, no principle has been described that would allow the volume of the regions to be chosen to sensibly balance the two competing requirements: small regions to reduce the error in assuming the probability is constant throughout the region and large regions so that counting-based estimates are robust. There are two ways in which these difficulties will need to be addressed: theoretically, in demonstrating that the estimate converges under sensible conditions, and practically, in demonstrating that the estimate is accurate and not overly sensitive to the choice of volumes for the sorts of data that are likely to be of interest. Hopefully, the simplicity of the approach described here will aid further development.
